# An novel role of sphingosine kinase-1 (SPHK1) in the invasion and metastasis of esophageal carcinoma

**DOI:** 10.1186/1479-5876-9-157

**Published:** 2011-09-22

**Authors:** Jian Pan, Yan-Fang Tao, Zhuan Zhou, Bang-rong Cao, Shui-Yan Wu, Yan-Lan Zhang, Shao-Yan Hu, Wen-Li Zhao, Jian Wang, Guo-Liang Lou, Zhen Li, Xing Feng, Jian Ni

**Affiliations:** 1 Department of Hematology and Oncology, Children's Hospital of Soochow University, Suzhou, China; 2 Department of Cell and Molecular Biology, Cancer Institute (Hospital), Chinese Academy of Medical Sciences, Peking Union Medical College, Beijing, China; 3 Hillman Cancer Center Lab, Department of Pathology, Pittsburgh University, G21 5117 centre Ave. Pittsburgh, PA 15206, USA; 4 Translational Research Center, Second Hospital, The Second Clinical School, Nanjing Medical University, Nanjing, China; 5 State Key Laboratory of Molecular Oncology, Cancer Institute (Hospital), Peking Union Medical College, Chinese Academy of Medical Sciences, Beijing 100021, China; 6 Translational Research Center, Chang Hai Hospital, The Second Military Medical University, Shanghai, China; 7 Department of Clinical Pharmacology, Changhai Hospital, Second Military Medical University, 168 Changhai Road, Shanghai 200433, China

## Abstract

**Background:**

Treatment failure for esophageal carcinoma is frequently due to lymph node metastasis and invasion to neighboring organs. The aim of the present study was to investigate invasion- and metastasis-related genes in esophageal carcinoma cells *in vitro *and *in vivo*.

**Methods:**

A metastasis model using a Matrigel invasion clonal selection approach was employed to establish a highly invasive subline EC9706-P4 from the esophageal carcinoma cell (ESCC) line EC9706. The differentially expressed genes of the subline and the parental cells determined by gene microarrays were further analyzed by RT-PCR and Western blotting.

**Results:**

We identified *sphingosine kinase 1 (SPHK1) *as an invasion and metastasis-related gene of esophageal cancer. *SPHK1 *was overexpressed in the EC9706-P4 subline with high invasive capacity. Among six ESCC lines tested, KYSE2 and KYSE30 cells showed the highest *SPHK1 *mRNA and protein expressions as well as the most invasive phenotype. By Western blotting, in 7/12 cases (58%), SPHK1 expression was higher in esophageal carcinomas than in the companion normal tissue. In 23/30 cases (76%), SPHK1 protein expression was upregulated in the tumors compared to matched normal tissue by immunohistochemistry (IHC). Esophageal carcinoma tissue microarray analysis indicated that SPHK1 expression correlated with the depth of tumor invasion (*P *< 0.0001) and lymph node metastasis (*P *= 0.016). By Kaplan-Meier analysis, strong SPHK1 expression was significantly associated with clinical failure (*P *< 0.01), suggesting the involvement of SPHK1 in aggressiveness of human esophageal carcinoma. *SPHK1 *overexpression significantly increased the invasiveness of EC9706 cells *in vitro *and also increased EC9706 cell growth and spontaneous metastasis *in vivo*, promoting significant increases in tumor growth, tumor burden and spontaneous lung metastasis in nude mice. *SPHK1 *expression significantly correlated with the expression of many EGFR pathway genes associated with invasion of cancer cells. SPHK1 protein expression also significantly correlated with the phosphorylation of EGFR.

**Conclusion:**

In summary, our data implicate *SPHK1 *in the metastasis of esophageal cancer. Our study also identified downstream mediators of SPHK1 in esophageal cancer cells that may mediate enhanced malignant behavior, and several of these mediators may be useful as therapeutic targets.

## Introduction

Human esophageal carcinoma, one of the most common causes of cancer death worldwide, occurs at a very high frequency in China [1,2]. Esophageal carcinomas often have poor prognosis due to early lymph node metastasis and invasion of neighboring organs such as the aorta, trachea, bronchus, pericardium and lung [2]. Therefore, disrupting the aggressive metastatic phenotype is essential for developing an effective treatment for esophageal cancer. Although several molecules have been reported to contribute to the ability of esophageal carcinoma cells to metastasize and invade normal tissue, such as N-cadherin [3], TSLC1 [4] and MTA1 [5], the underlying mechanism remains obscure. Considering the complexity of tumor invasion and metastasis, various experimental approaches have been developed to systematically identify genes that are involved in the process. By directly comparing the differentially expressed genes between liver metastatic and primary tumor tissues, *POSTN*, encoding the periostin protein, was identified as a gene associated with colon cancer and liver metastasis [6]. Cancer metastasis is thought to originate from a small proportion of cancerous cells in primary tumors. Therefore, screening for a subpopulation of cells with high metastatic potential from a parent tumor cell line in experimental models is a well-defined method for discovering genes that play roles in metastasis, especially that which preferentially occurs in specific organs. For example, microarray analysis of sublines of the MDA-MB-231 cell line with high lung or bone metastatic selection in nude mice led to identification of a set of genes that mark or mediates breast cancer metastasis in these tissues [7-9].

In order to derive a subpopulation of cells with high metastatic potential from tumor cell lines, we have established a model system to inspect genes involved in different steps of metastasis including invasion, survival and arrest. We screened for and selected an esophageal tumor cell subline with high invasive potential and analyzed genes which may correspond to this phenotype by gene microarray. Through this analysis, we identified *sphingosine kinase 1 (SPHK1*) as one such gene that participates in esophageal carcinoma invasion and metastasis.

SPHK1 is a conserved lipid kinase that catalyzes formation of important regulators of inter- and intracellular signaling. It is a ubiquitously expressed, evolutionary conserved enzyme that catalyzes phosphorylation of sphingosine (Sph) and dihydrosphingosine (dhSph) to sphingosine 1-phosphate (S1P) and dhS1P, respectively. SPHK1 is transiently activated in response to a large variety of agonists and has been shown to contribute to signaling cascades elicited by TNF-α [10], VEGF and 17β-estradiol [11]. Accordingly, SPHK1 can mediate biological effects of TNF-α such as induction of COX2 and generation of PGE2 in fibroblastic cells and MCP-1 in endothelial cells [12].

Of interest is that *SPHK1 *mRNA is frequently overexpressed in a variety of solid tumors, suggesting an important role for its encoded enzyme during tumorigenesis. Overexpression of *SPHK1 *is thought to be oncogenic and renders transfected cells chemoresistant. Bonhoure *et al*. [13] reported that targeting *SPHK1 *overcomes the multi-drug- resistant gene (MDR)-associated chemoresistance of HL60 cells. The oncogenic properties of *SPHK1 *have been demonstrated both *in vitro *and *in vivo *in experimental models based on its overexpression in NIH3T3 cells [14]. Overexpression of *SPHK1 *facilitates anchorage-independent growth *in vitro *and leads to tumor formation in SCID mice. Furthermore, more recent studies have demonstrated that phosphorylation of SPHK1 and subsequent membrane translocation are required for its pro-oncogenic function. Overexpression of *SPHK1 *up-regulates *MMP1 *mRNA and promoter activity as well as its protein levels, and this action by *SPHK1 *requires activation of the ERK1/2-Ets1 and NF-kB pathways [12]. In addition, high levels of the SPHK1 enzyme and its downstream product, S1P, correlate with poor survival of glioblastoma patients [15,16], and this unusual enhancement of neural cell invasion requires upregulation of the matricellular protein CCN1/Cyr61. These data suggest that enhanced SPHK1 activity in glioblastoma may drive the invasive potential of these cells.

Few studies by other investigators have specifically analyzed the effect of SPHK1 on invasiveness of cancer cells. Our report is the first to implicate the involvement of *SPHK1 *in the metastasis of esophageal cancer. We observed the overexpression of *SPHK1 *transcripts in esophageal carcinoma and identified downstream mediators that may mediate enhanced malignant behavior in these tumor cells. As several of these mediators may be useful as therapeutic targets of esophageal carcinoma, our findings will have implications for cancer drug development.

## Materials and methods

### Cell culture conditions

The esophageal carcinoma cell (ESCC) line EC9706 (a gift from Dr. Minrong Wang, Chinese Academy of Medical Sciences Cancer Institute Hospital, Peking University Medical School, Beijing, China) was cultured in RPMI 1640 supplemented with 10% fetal bovine serum (FBS). Other ESCC lines (KYSE30, KYSE150, NEC, KYSE510, and KYSE2) were generously provided by Dr.Y. Shimada, Kyoto University.

### Selection of invasive sublines from EC9706 cells

To select a highly invasive subpopulation, EC9706 cells were seeded on a Matrigel (Becton Dickinson, Franklin Lakes, NJ) coated, 8 μm-pore transwell (Costar, Cambridge, MA). 24 hours later, cells that had invaded to the other side of the transwell membrane were collected, expanded and then re-seeded into another Matrigel coated transwell. Such selection rounds for highly invasive cells were repeated four times, resulting in a subline from each generation designated as EC9706-P1, EC9706-P2, EC9706-P3 and EC9706-P4.

### Quantitative RT-PCR

QRT-PCR was performed as my previous manuscript described [17-20], briefly cells were harvested in Trizol^® ^reagent (Invitrogen, Carlsbad, CA), and total RNA was isolated according to the manufacturer's instructions. Single-stranded cDNA was synthesized from 4 μg total RNA using M-MLV reverse transcriptase (Invitrogen), with an oligo(dT) 18-mer as the primer, in a final reaction volume of 25 μl. Amplifications of specific transcripts from the cDNA were performed using the following primers: *SPHK1 *sense 5'-ACAGTGGGCACCTTCTTTC-3', antisense 5'-CTTCTGCACCAGTGTAGAGGC -3'; *SPHK2 *sense *5'-GCACGGCGAGTTTGGTTC-3'*, antisense *5'-GAGACCTCATCCAGAGAGACTAG-3'; TM4SF3 *sense 5'-CCAAACCCAGTATCTCA-3', antisense 5'- GACAAGCCTGTAACGAA-3'; *Integrin α6 *sense 5'- GCC TTG CAC GAT GAT ATG GAG-3', antisense 5'- GAT GAG CTG TCT GGAGAA-3'; *Integrin β4 *sense 5'- GCATCGTGGTCATGG AGAGCAG-3', antisense 5'- AATGTCCCTCGTGCACACAGC-3'; *MET *sense 5'- CATGCCGACAAGTGCAGTA-3', antisense 5'- TCTTGCCATCATTGTCCAAC-3'; *GAPDH *sense 5'- TGGTATCGTGGAAGGACTCATGAC-3', antisense 5'- ATGCCAGTGAGCTTCCCGTTCAGC-3'; *ADAM12m *sense 5'- GCACCTCCCTTCTGTGACAAGTTT-3', antisense 5'- CTTGGTGTGGATATTGTGGAGCAG-3'; *CLDN1 *sense 5'- GCGCGATATTTCTTCTTGCAGG-3', antisense 5'- TTCGTACCTGGCATTGACTGG-3'; *IGFBP1 *sense 5'- ATCTGATGGCCCCTTCTGAA-3', antisense 5'- AGCCTTCGAGCCATCATAGGTA-3'; *A2M *sense 5'- ACCAGGACGATGAAGACTGCAT-3', antisense 5'- CCACGAATCACAGAGTAAGGCA-3'; *Integrin α3 *sense 5'- AAGCCAAGTCTGAGACT-3', antisense 5'- GTAGTATTGGTCCCGAGTCT-3'; *TFPI2 *sense 5'- CGCCGGATCCTTTCTCGGAC-3', antisense 5'- GAATGTCTCGAGTTGCTTCTTCCGA-3'; *LOXL2 *sense 5'- GGCCGCCACGCGTG GATC-3', antisense 5'- CCCAAGGGTCAGGTAGCAGCCCC-3'; *CYR61 *sense 5'- AGCCCAACTGTAAACATCAG-3', antisense 5'- CATCCAGCGTAAGTAAACCT-3'; *TIMP3 *sense 5'- TTCGGTTACCCTGGCTACCA-3', antisense 5'- CTGCAG TAGCCG CCTTCT-3'.

### Tissue samples and IHC

Samples were obtained from patients who attended Chinese Academy of Medical Sciences Cancer Hospital from January 2001 to December 2005. Ethical approval was provided by the Chinese Academy of Medical Sciences Cancer Hospital Ethics Committee. None of the patients received any neoadjuvant therapy prior to surgery. Prior patient consent and approval from the Institute Research Ethics Committee were obtained before we used these clinical materials for research purposes. Paired samples of fresh normal esophageal tissues and esophageal carcinoma tissues from the same patient were collected by the Department of Pathology in the Chinese Academy of Medical Sciences Cancer Hospital (Beijing, China). Each patient had received a pathologically and clinically confirmed diagnosis of esophageal squamous cell carcinoma. Primary tumor regions and the corresponding histologically normal esophageal mucosa were separated by experienced pathologists and immediately stored at -70°C until use. None of the patients received treatment before surgery.

The expression of SPHK1 in the esophageal tumors was determined by assessing its staining using tissue microarrays from 154 clinical cases, of which 124 of the esophageal cancer specimens had clinical follow-up records, these patients were followed up 8 years and 117 patients died at the end-points. In addition, 30 of these specimens had paired normal epithelia. For immunostaining of SPHK1, a DAKO CSA kit was used. The anti-SPHK1 antibody (ab16491, Abcam, 10 μg/ml) was incubated with the sectioned tissues for 1 h in citrate buffer. After staining, the slides were evaluated by two pathologists. SPHK1 expression was determined by scoring intensity and percentage of staining. Tissues with no staining were rated as 0; those with faint staining or moderate to strong staining in < 25% of cells as 1; with moderate staining or strong staining in 25-50% of cells as 2; and with strong staining in > 50% of cells as 3.

### Immunofluorecence

Cells were fixed in 4% paraformaldehyde and then permeablized with 0.2% Triton X-100. Coverslips were blocked with 10% non-immune sheep serum. Primary antibodies were diluted in 1% bovine serum albumin/PBS and incubated overnight at 4°C. The antibody against SPHK1 (ABIN552700, poly rabbit antibody, 1:50) was obtained from http://antibodies-online.com. The secondary antibody was Cy5-conjugated goat anti-rabbit IgG (Jackson Immunoresearch Laboratories Inc., West Grove, PA). Coverslips were mounted on glass sides with 0.5 μg/ml DAPI/fluorescence protection agent. Images were acquired with an Olympus confocal microscope.

### Generation of *SPHK1 *overexpressing cells

The human *SPHK1 *cDNA was cloned into the pcDNA4.0 expression vector (Invitrogen) containing a c-myc epitope at the C-terminus to give the pcDNA4.0-SPHK1-c-myc vector. The SPHK1 expression vector was transfected into EC9706 cells using Lipofectamine 2000 (Invitrogen), according to the manufacturer's recommendations. Cells carrying the recombinant SPHK1 or empty (control) vector were selected by culturing in the presence of 200 ng/ml Zeocin (Invitrogen) for more than 4 weeks. Two *SPHK1 *stably transfected EC9706 cell clones (C17, C24) and one *SPHK1 *negative clone (C7) were chosen for subsequent experiments. Transfection of duplex siRNAs was performed in the same manner. The *SPHK1 *siRNA sequences as used before [21] were 5'-GGGCAAGGCCUUGCAGCUCd(TT)-3' and 5'-GAGCUGCAAGGCCUUGCCCd(TT)-3', control siRNA 5'-UUCUCCGAACGUGUCACGUd(TT)-3'.

### Invasion assays

Invasion assays were carried out similarly to the procedure for selecting invasive cell lines described above [17]. Briefly, 1 × 10^4 ^cells were placed in a 24-well transwell unit on polycarbonate filter with 8 μm pores coated with Matrigel. After a 24-h incubation period, the cells that had passed through the filter into the lower wells were stained, counted and photographed. All experiments were performed in triplicate and repeated twice.

### Western blot analysis

The proteins were separated by sodium dodecyl sulfate-polyacrylamide gel electrophoresis and then transferred to polyvinylidene difluoride membranes (Millipore, Bedford, MA). Blots were blocked and then probed with antibodies against the tag of SPHK1 protein c-myc (9E10 1:3000, Sigma, St. Louis, MO) or directlly against the SPHK1 protein (1 μg/ml, ab16491, Abcam), β-actin (1:5000, Sigma, St. Louis, MO), phospho-EGFR (Y1068,1:1000, Invitrogen), EGFR(1:1000, Invitrogen). After washing, the blots were incubated with horseradish peroxidase-conjugated secondary antibodies and visualized using an enhanced chemiluminescence kit (Pierce, Rockford, IL).

### Xenograft assays in nude mice

Female *nu/nu *mice, obtained from the Jackson Laboratory (Vitalriver, China), were kept in a specific pathogen-free facility at the Experimental Center of the Chinese Academy of Medical Sciences, Beijing, China. We are ensure animals used for scientific purposes are treated and cared for ethically and humanely. Female mice, aged 4-6 weeks, were used in these experiments. Parental EC9706, *SPHK1 *transfected EC9706 clones C17 and C24, or vector transfected cells were each subcutaneously injected into eight nude mice. Three months after the injection, the mice were sacrificed and examined for subcutaneous tumor growth and metastasis development. The tumor volumes were calculated according to the following formula: volume = length × width^2^/2. After the last treatment, the mice were sacrificed, and the tumor volumes and weights were measured. Xenografts were detected using the anti-SPHK1 antibody (10 μg/ml, ab16491, Abcam) with each section examined.

### Statistical analysis

All data are presented as mean ± standard deviation (SD). Statistical analysis was performed using the Statistical Package for the Social Sciences (SPSS) (SPSS Inc., Chicago, IL). Student's two-tailed *t*-test was used to compare the groups. *P *≤ 0.05 was considered significant.

## Results

### *SPHK1 *is overexpressed in the EC9706-P4 subline with high invasive capacity

**Figure 1:**
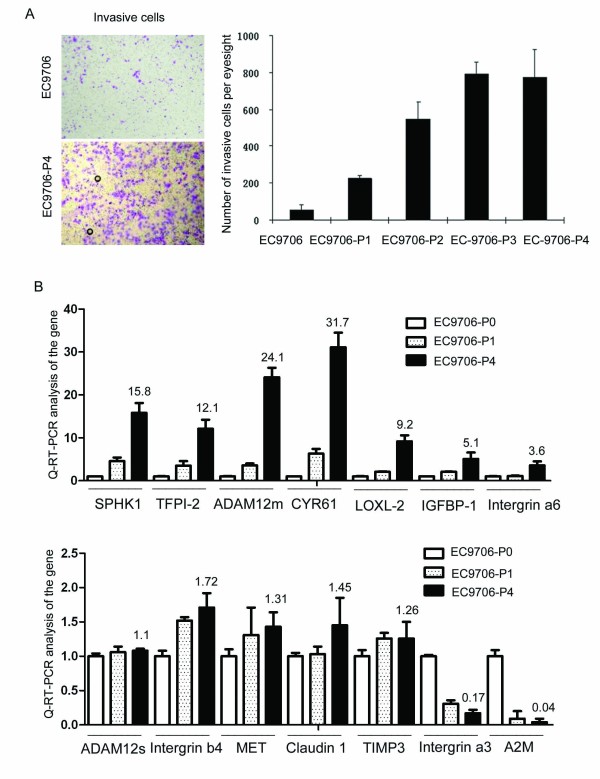
**Overexpression of *SPHK1 *in the esophageal carcinoma EC9706-P4 subline with high invasive capacity**. EC9706 cells were seeded onto Matrigel-coated transwells and allowed to invade into the lower chamber of the transwell, collected, expanded and then re-seeded onto another Matrigel-coated transwell. After four rounds of selection, the relatively stable, highly invasive subline, EC9706-P4, was established. (A) Transwell invasion analysis showed the invasive ability of EC9706-P4 was about 15-fold greater than the parent cell line EC9706 (*P *< 0.05) (B) The differential gene expression profiles of EC9706 and EC9706-P4 were analyzed by microarray, and expression of some genes were confirmed by QRT-PCR. Most of the dysregulated genes were previously associated with tumor invasion and metastasis. Notably, *SPHK1 *expression gradually increased through each round of selection from EC9706 to EC9706-P4.

To establish an esophageal carcinoma invasion model, EC9706 cells were seeded onto Matrigel-coated transwells. The cells were allowed to invade into the lower chamber of the transwell, collected, expanded and then re-seeded onto another Matrigel-coated transwell. Cells were harvested after four rounds of selection, resulting in the establishment of a relatively stable, highly invasive subline, EC9706-P4. The invasive ability of this line was about 15-fold greater than that of the parent cell line, EC9706, in matrigel invasion tests, but equal to that of the third selection round line, EC9706-P3 (Figure 1A), suggesting that the metastatic potential of the sublines reached a plateau by the fourth round of selection. Thus, by Matrigel invasive subpopulation selection, we were able to establish the highly invasive and metastatic esophageal carcinoma cell line EC9706-P4.

The gene expression profiles of EC9706 and EC9706-P4 were then analyzed by microarrays. Between these cell lines, 124 genes were differentially expressed, including 71 upregulated genes and 53 downregulated genes in EC9706-P4 relative to EC9706 (cutoff, fold ≥ 2.0, Additional file 1). Most of the dysregulated genes have been previously shown to be involved in tumor invasion and metastasis, such as TFPI-2 [17], LOXL2 [22-24], ADAM12m [25,26], IGFBP1 [27,28], *integrin α6*[29,30], *CD44 *[31]and *CYR61 *[32]. Expression of some of these genes were subsequently tested by QRT-PCR (Figure 1B). One of these genes, *SPHK1*, was of particular interest to our study. Trojanowska *et al*. showed that overexpression of *SPHK1 *up-regulated *MMP1 *mRNA and promoter activity as well as its protein levels [12], and the *MMP1 *gene is known to play a very important role in the invasion of esophageal cancer. We found by QRT-PCR analysis that *SPHK1 *expression gradually increased with each round of selection from the EC9706 parent cells to the highly invasive EC9706-P4 subline (Figure 1B).

### *SPHK1 *is upregulated in esophageal carcinoma and correlates with invasion and clinical outcome

**Figure 2:**
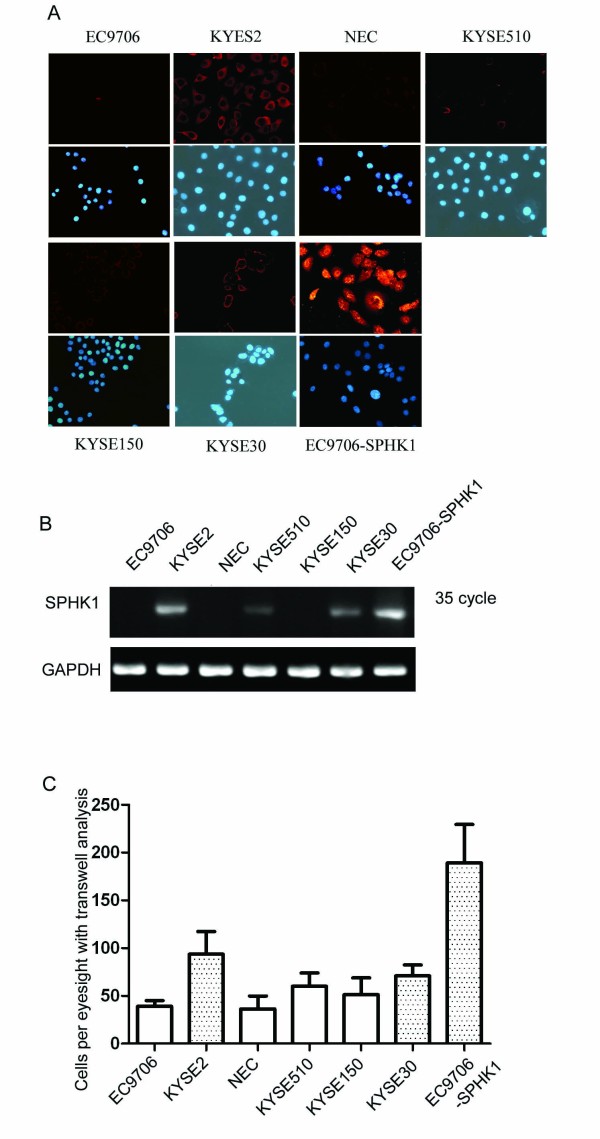
**Correlation of *SPHK1 *expression with invasiveness of esophageal carcinoma cell lines**. (A, B) *SPHK1 *expression was analyzed by RT-PCR and immunofluorecence in the esophageal carcinoma cell lines: KYSE30, KYSE150, KYSE510, KYSE2, EC9706 and NEC. (B) In these cell lines, *SPHK1 *was detected by PCR after 35 cycles. (C) Invasion assays were carried out by seeding 1 × 10^4 ^cells in a 24-well transwell unit on polycarbonate filter with 8-μm pores coated with Matrigel. After a 24-h incubation period, the cells that had passed through the filter into the lower wells were stained, counted and photographed. All experiments were performed in triplicate and repeated twice. KYSE2 and KYSE30 cells showed the highest SPHK1 expression at the mRNA and protein levels, and they were also the most invasive among the six cell lines.

**Figure 3:**
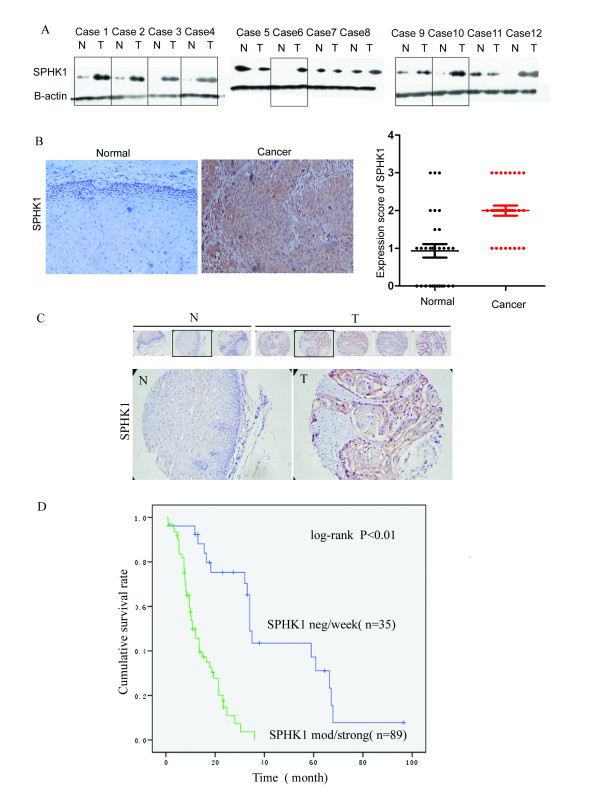
**Upregulation of SPHK1 in esophageal carcinoma and correlation with clinical outcome**. SPHK1 protein expression was assessed in esophageal carcinoma tissues. (A) Twelve sample pairs, each consisting of esophageal carcinoma tissue and normal esophageal tissue from the same patient, were analyzed by Western blotting. SPHK1 was overexpressed in seven of twelve esophageal carcinoma tissues (58%) compared to the companion normal tissue. (B) The expression SPHK1 in 30 pairs of esophageal-carcinoma and normal tissues were also analyzed by IHC. In 23 of 30 cases (76%), *SPHK1 *expression appeared to be upregulated in the tumors when compared to the companion normal tissue with the following immunostaining scores: normal: 0.93 ± 0.173; tumor: 2.06 ± 0.46, *P *< 0.01. (C-D) SPHK1 protein expression in an esophageal carcinoma tissue microarray (n = 274 tissue microarray elements from 124 cases of esophageal squamous carcinoma). By Kaplan-Meier analysis, strong SPHK1 expression (staining intensity equal to, or greater than, 2) was significantly associated with clinical failure, whereas the weak SPHK1staining (staining intensity less than 2) was found in individuals with delayed clinical failure, *P *< 0.01.

*SPHK1 *expression was analyzed by RT-PCR and immunofluorecence in esophageal carcinoma cell lines, including KYSE30, KYSE150, KYSE510, KYSE2, EC9706, and NEC cells (Figure 2A). In these cell lines, *SPHK1 *was detected by PCR after 35 cycles (Figure 2B). KYSE2 and KYSE30 cells showed the highest *SPHK1 *expression at both the mRNA and protein levels, and they were also the most invasive lines among the six cell lines tested (Figure 2C). To assess SPHK1 protein expression in esophageal carcinoma tissues, 12 paired samples, each consisting of esophageal carcinoma tissue and normal esophageal tissue from the same patient, were analyzed by Western blotting. SPHK1 protein was found to be overexpressed in seven of the twelve (58%) esophageal carcinoma tissues; that is, SPHK1 expression was higher in the tumor tissue than in the companion normal tissue (Figure 3A). We then extended this analysis by performing immunohistochemistry (IHC) on 30 pairs of esophageal-carcinoma and normal tissues. In 23 cases (76%), SPHK1 expression appeared to be upregulated in the tumors when compared to the companion normal tissue with the following immunostaining scores: normal: 0.93 ± 0.173; tumor: 2.06 ± 0.46, *P *< 0.01 (Figure 3B). We then evaluated the expression of SPHK1 protein in an esophageal carcinoma tissue microarray (n = 274 tissue microarray elements from 124 cases of esophageal squamous carcinoma) (Figure 3C). The expression rates of SPHK1 in cases from tussue microarrays is that 18/124 (14.5%) cases with no SPHK1 expression, 17/124(13.7%) cases with SPHK1 expression score 1, 32/124(25.8%) cases with SPHK1 expression score 2 and 57/124(46.0%) cases with SPHK1 score 3. Statistical analysis indicated that in the esophageal carcinoma, the SPHK1 expression correlated with the depth of tumor invasion (*P *< 0.0001) and lymph node metastasis (*P *= 0.016) (Table 1). There was no significant association with age, sex and tumor differentiation.

**Table 1:** SPHK1 correlated with depth of tumor invasion and lymph node metastasis in esophageal carcinoma

Variables	Score of SPHK1 expression				*P *value
	**0**	**1**	**2**	**3**	

Age, year					0.374
Mean	61.7	61.2	63.2	60.1	
SD	11.0	12.8	11.9	14.3	
Gender					0.494
Male	7	11	15	26	
Female	11	6	17	31	
pT (primary tumor)					< 0.0001*
pT0	0	5	4	0	
pT1	1	2	6	6	
pT2	6	4	10	11	
pT3	11	6	12	40	
pN(lymphnode metastasis)					0.016*
pN0	14	10	16	17	
pN1	4	7	16	40	
Differentiation					0. 737
high	4	15	9	19	
moderate	6	2	12	21	
low	8	0	11	17	

Expression of SPHK1 protein was analyzed in 124 cases of esophageal squamous carcinoma. After staining, slides were evaluated by two pathologists. SPHK1 expression was determined by scoring intensity and percentage of cell staining. Tissues with no staining were rated as 0; those with faint staining or moderate to strong staining in < 25% of cells as 1; with moderate staining or strong staining in 25-50% of cells as 2; and with strong staining in > 50% of cells as 3. Statistical analysis indicated that in the esophageal carcinoma, SPHK1 expression was correlated with depth of tumor invasion (*P *< 0.0001) and lymph node metastasis (*P *= 0.016). There was no significant association with age, sex or tumor differentiation.

We therefore examined whether expression of the SPHK1 protein could be used to predict clinical outcome in esophageal carcinoma patients with available survival information. By Kaplan-Meier analysis, strong SPHK1 expression (staining intensity ≥ 2) was significantly associated with clinical failure, whereas the weak SPHK1 staining (staining intensity less than 2) was found in individuals with delayed clinical failure (*P *< 0.01) (Figure 3D). These findings further suggest that SPHK1 overexpression is significantly associated with aggressive human esophageal carcinoma.

### *SPHK1 *upregulates esophageal carcinoma invasive and spontaneous metastasis potential

**Figure 4:**
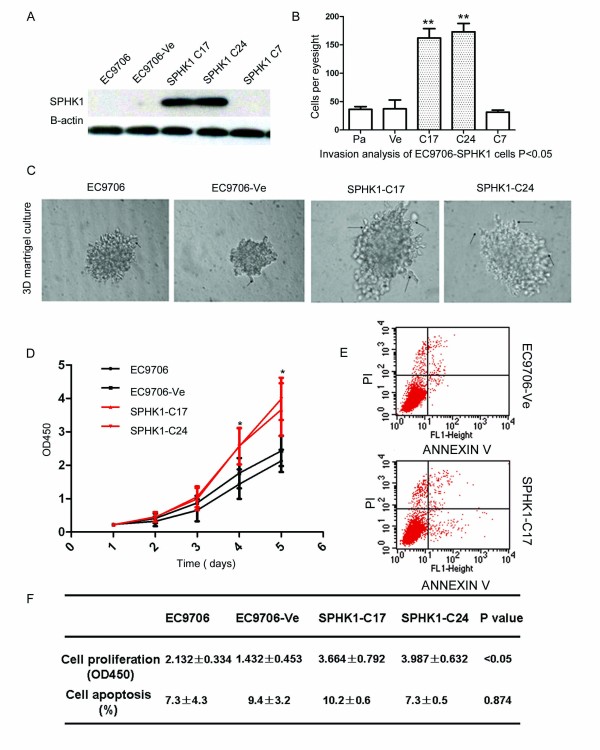
***SPHK1 *overexpression increased invasive potential of esophageal carcinoma cells**. We assessed the impact of gene transfection-mediated *SPHK1 *upregulation in EC9706 cells. (A) The two stable expression clones SPHK1-C17 and SPHK1-C24 both had markedly upregulated *SPHK1 *expression in EC970 cells. (B) In the transwell invasion assay, *SPHK1 *overexpression significantly increased the invasion of EC9706 cells. The numbers of invaded cells in the *SPHK1 *overexpressing cells (SPHK1-C17 group = 162.0 ± 16.52; SPHK1-C24 group = 173.0 ± 14.73) were higher compared with that of the vector group (37.33 ± 15.57), EC-9706 group (36.33 ± 4.933) and SPHK1-C7 group (31.33 ± 3.786); ANOVA *P *< 0.0001. (C) In 3D Matrigel culture assays, *SPHK1 *overexpression significantly increased the diameter and the invasive morphology of EC9706 cell clones. (D, F) *SPHK1 *overexpression significantly increased the proliferation of EC9706 cells. The OD450 values of the SPHK1-C17 group (3.664 ± 0.792) and *SPHK1*-C24 group (3.987 ± 0.632) were higher than that of the vector group (1.432 ± 0.453) and EC-9706 group (2.132 ± 0.334), ANOVA *P *< 0.031. (E, F) Annexin V assay showed overexpression of the *SPHK1 *gene did not influence the apoptosis level of EC9706 cells. The percentages of apoptotic cells in the SPHK1-C17 group (10.2 ± 0.2) and SPHK1-C24 group (7.3 ± 0.5) were comparable to that of the vector group (9.4 ± 3.2) and EC9706 group (7.3 ± 4.3), ANOVA *P *= 0.874.

**Figure 5:**
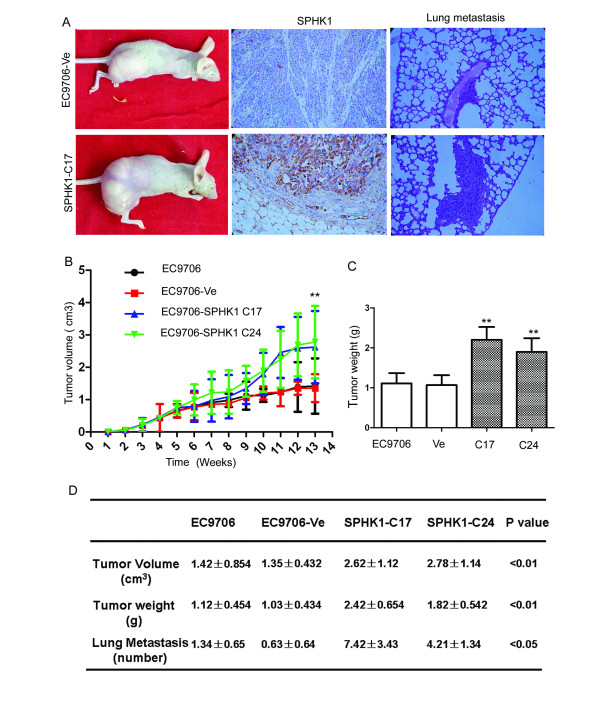
***SPHK1 *overexpression increased esophageal carcinoma spontaneous metastasis potential**. *SPHK1 *overexpression increased EC9706 cell growth and spontaneous metastasis in nude mice. Several stable transfected clones were screened and two of them, (A) *SPHK1*-C17 and *SPHK1*-C24 were tested in animal experiments. (B, C) The *SPHK1 *gene promoted tumor growth and tumor burden in nude mice. *SPHK1 *significantly promoted growth of EC9706 xenografts (SPHK1-C17: 2.62 ± 1.12 cm^3^; SPHK1-C24: 2.78 ± 1.14 cm^3^) compared to transfected control (EC9706: 1.42 ± 0.854 cm^3^) or control EC9706 cells (EC9706-Ve: 1.35 ± 0.432 cm^3^, ANOVA *P *< 0.01, B and D). Expression of *SPHK1 *increased weight of tumors (SPHK1-C17: 2.42 ± 0.654 g; SPHK1-C24: 1.82 ± 0.542 g) compared to transfected control (EC9706: 1.12 ± 0.454 g) or control EC9706 cells (EC9706-Ve: 1.03 ± 0.434 g, ANOVA *P *< 0.01, C and D). We also observed that *SPHK1 *upregulation dramatically increased spontaneous lung metastasis of EC9706 cells (SPHK1-C17:7.42 ± 3.43 cm^3^; SPHK1-C24: 4.21 ± 1.34 cm^3^) compared to transfected control (EC9706: 1.34 ± 0.65 cm^3^) or control EC9706 cells (EC9706-Ve: 0.63 ± 0.064 cm^3^, ANOVA *P *< 0.05, A and D).

We next assessed the impact of transfection-mediated *SPHK1 *gene upregulation in EC9706 cells. Several *SPHK1 *stable transfected clones were screened and two of them, SPHK1-C17 and SPHK1-C24, had markedly upregulated expression of *SPHK1 *in EC970 cells (Figure 4A). In the transwell invasion assay, upregulation of *SPHK1 *significantly increased the invasiveness of EC9706 cells (Figure 4B). In the 3D Matrigel culture assays, *SPHK1 *overexpression significantly increased the diameter and the invasive morphology (Figure 4C) as well as the proliferation (Figure 4D, F) of the EC9706 cell clones, but did not influence their apoptosis levels in an Annexin V assay (Figure 4E, F). We also examined whether *SPHK1 *could increase EC9706 cell growth and spontaneous metastasis in nude mice. The SPHK1-C17 and SPHK1-C24 stable overexpressing clones were tested in animal experiments and showed that the *SPHK1 *gene promoted tumor growth and tumor burden in nude mice. *SPHK1 *significantly promoted growth of EC9706 xenografts (SPHK1-C17: 2.62 ± 1.12 cm^3^; SPHK1-C24: 2.78 ± 1.14 cm^3^) compared to transfected control (EC9706: 1.42 ± 0.854 cm^3^) or control EC9706 cells (EC9706-Ve: 1.35 ± 0.432 cm^3^, ANOVA *P *< 0.01 Figure 5B, D). Expression of *SPHK1 *increased weight of tumors (SPHK1-C17: 2.42 ± 0.654 g; SPHK1-C24: 1.82 ± 0.542 g) compared to transfected control (EC9706: 1.12 ± 0.454 g) or control EC9706 cells (EC9706-Ve: 1.03 ± 0.434 g, ANOVA *P *< 0.01 Figure 5C, D). We also observed that *SPHK1 *upregulation dramatically increased spontaneous lung metastasis of EC9706 cells (SPHK1-C17:7.42 ± 3.43 cm^3^; SPHK1-C24: 4.21 ± 1.34 cm^3^) compared to transfected control (EC9706: 1.34 ± 0.65 cm^3^) or control EC9706 cells (EC9706-Ve: 0.63 ± 0.064 cm^3^, ANOVA *P *< 0.05 Figure 5A, D). These studies support the view that *SPHK1 *expression is vital for the maintenance of invasive and metastatic potential of esophageal carcinoma cells.

### SPHK1 promotes metastasis *via *activation of the EGFR pathway

**Figure 6:**
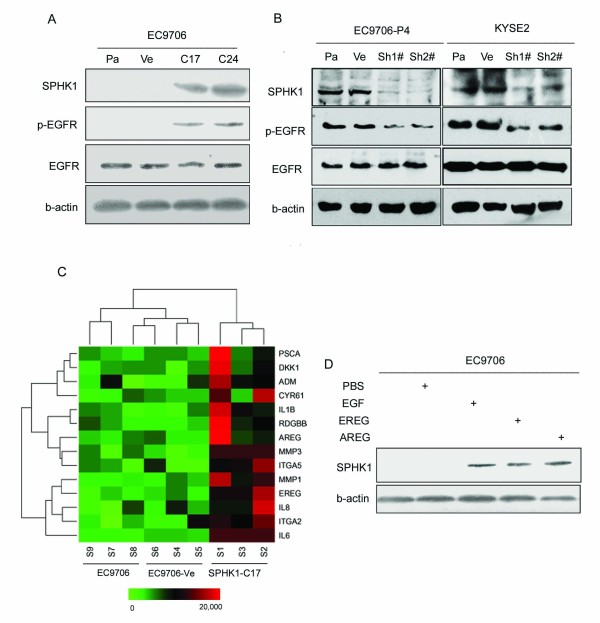
***SPHK1 *promoted metastasis *via *activation of the EGFR pathway**. To glean mechanistic insight into the role of *SPHK1 *in invasion and metastasis, we surveyed potential links between *SPHK1 *and key molecules of known relevance to invasion. (A) Western blotting indicated that the expression of SPHK1 was significantly correlated with the phosphorylation of EGFR. (B) Correspondingly, *SPHK1 *RNAi decreased the phosphorylation of EGFR in EC9706-P4 and KYSE2 cells. (C) We clustered the upregulated genes in the *SPHK1 *overexpression clones compared with control cells. Expression of *SPHK1 *was significantly correlated with the expressions of *IL6, ITGA2, IL8, EREG, MMP1, ITGA5, MMP3 *and *AREG*. Most of the genes are the downstream genes of EGFR pathway, and most of them have been associated with the invasion of cancer cells. (D) Western blotting indicated that the ligands of EGFR (EGF, EREG, and AREG) induced the expression of SPHK1 protein.

To glean mechanistic insight into the role of SPHK1 in invasion and metastasis, we surveyed potential links between this enzyme and key molecules of known relevance to invasion. Western blot analysis indicated that the expression of SPHK1 significantly correlated with the phosphorylation of EGFR, while knockdown of SPHK1 by RNAi decreased EGFR phosphorylation in EC9706-P4 and KYSE2 cells (Figure 6A, B). From the microarray analysis, we clustered the genes upregulated in the SPHK1 overexpression clones compared with control cells (Figure 6C). Expression of *SPHK1 *was significantly correlated with that of genes such as *IL6, ITGA2, IL8, EREG, MMP1, ITGA5, MMP3 *and *AREG*. Most of these genes are the downstream genes of the EGFR pathway, and most of them such as EREG [33,34], MMP1 [35] and AREG [33,34] have been reported to be associated with the invasion of cancer cells. Western blotting indicated that the ligands of EGFR, EGF, EREG and AREGinduced the expression of the SPHK1 protein (Figure 6D).

## Discussion

Metastasis is a multi-factorial process, including tumor cells capable of escaping their normal microenvironment, traversing into and out of lymphatic or blood vessels and proliferating in new ''soil'' [36]. Implicit in these stages, invasion is the critical ability for tumor cells to metastasize [36]. During invasion, malignant cells reside on or within two major types of extracellular matrices, the basement membrane and the stromal matrix [37]. The basement membrane is one of the most important barriers against cancer cell invasion [37]. Therefore, for this study, we used Matrigel, a solubilized basement membrane preparation from the Engelbreth-Holm-Swarm (EHS) mouse sarcoma, as a model basement matrix to mimic esophageal carcinoma invasion *in vivo*. Although EC9706, an esophageal squamous carcinoma cell line, can invade and form spontaneous lung metastasis nodules in *nu/nu *mice, its metastatic potential is relatively low [38]. The metastatic ability of EC9706 may arise from a few subclones with high metastatic potential among the parental cells. By screening with our *in vitro *model, the subline EC9706-P4 with high invasion potential was established. This subline also exhibited high spontaneous metastatic potential *in vivo*. Microarray analysis was used to determine which genes may be involved in invasion and metastasis. However, the microarray analysis of esophageal cancer tissues demonstrated that *SPHK1 *was significantly overexpressed in these tumor tissues, and that this expression significantly correlated with tumor invasion, lymph node metastasis and clinical stage, indicating that SPHK1 is involved in esophageal carcinoma invasion and metastasis. *SPHK1 *is up-regulated in many types of cancers and has been suggested as a potentially new therapeutic target. However, it is not yet known what signals the cancer cells use to apparently constitutively up-regulate expression of this enzyme, nor is it clear why it has such a profound role in tumorigenesis. A recent study examined the role of *SPHK1 *in intestinal tumorigenesis in the Min mouse in which intestinal adenomas develop spontaneously [39]. Deletion of the *SPHK1 *gene in these mice resulted in reduction of adenoma size. Concomitantly, epithelial cell proliferation in the polyps was attenuated, suggesting that *SPHK1 *regulates adenoma progression [39].

Exogenous expression of *SPHK1 in vitro *and *in vivo *further showed that it is a key factor in esophageal carcinoma cell invasion. In the transwell invasion assay, upregulation of *SPHK1 *expression significantly increased the invasion of EC9706 cells. Furthermore, upregulation of *SPHK1 *expression significantly increased the proliferation of EC9706 cells *in vitro *as well as increased EC9706 cell growth and spontaneous metastasis in nude mice. These studies support the view that *SPHK1 *expression is vital for the maintenance of invasive and metastatic potential of esophageal carcinoma cells. Interestingly, neutralizing S1P, the product of SPHK1 enzymatic activity, with a specific monoclonal antibody was remarkably effective in slowing progression of cancers, such as lung [40], colon [41], breast [42,43], melanoma [44] and ovarian cancers [21,45] in murine xenograft and allograft models [46]. A critical question raised by these observations is how neutralization of this simple lysophospholipid can have such dramatic effects on tumor progression.

To glean mechanistic insight into the role of SPHK1 in invasion and metastasis, we surveyed potential links between SPHK1 and key molecules related to EGFR. Western blot analysis indicated that the expression of SPHK1 was significantly correlated with the phosphorylation of EGFR. In clustering the upregulated genes in the SPHK1 overexpression clones compared with control cells, SPHK1 expression was significantly correlated with that of IL6, ITGA2, IL8, EREG, MMP1, ITGA5, MMP3 and AREG. Western blotting indicated that the ligands of EGFR, EGF, EREG and AREG induced the expression of SPHK1 protein. These findings provide evidence for cross-talk between SPHK1 and the EGFR pathway and reveal a key role for SPHK1 in integrating events downstream of EGF receptors. An intriguing possibility is that many growth and angiogenic factors such as EGF and AREG involved in tumorigenesis may act through SPHK1 activation [47]. Because both EGF and AREG have been implicated in progression of esophageal cancer, it was of interest to examine the involvement of SPHK1. There are numerous reports of rapid and transient activation of SPHK1 by growth and angiogenic factors [48,49]) that stimulate its phosphorylation at Ser225 [50] and subsequent translocation to the plasma membrane [51] where its substrate sphingosine resides, resulting in local formation of S1P [52]. Some cross-talk models between EGFR and SPHK1/S1P have been proposed previously, *Estrada-Bernal, A et al*[53] have reported that treatment of glioma cell lines with EGF led to increased expression and activity of SphK1. Expression of EGFRvIII in glioma cells also activated and induced SphK1. In addition, siRNA to SphK1 partially inhibited EGFRvIII-induced growth and survival of glioma cells as well as ERK MAP kinase activation. SphK1 activity is necessary for survival of GBM-derived neurosphere cells, and EGFRvIII partially utilizes SphK1 to further enhance cell proliferation. *Shida, D. et al *[54]reported that LPA markedly enhanced SphK1 mRNA and protein in gastric cancer MKN1 cells, DLD1 colon cancer cells and MDA-MB-231 breast cancer cells. LPA transactivated the epidermal growth factor receptor (EGFR) in these cells, and the EGFR inhibitor AG1478 attenuated the increased SphK1 and S1P(3) expression induced by LPA. Their research finally showed that SphK1 is a convergence point of multiple cell surface receptors for three different ligands, LPA, EGF, and S1P, which have all been implicated in regulation of motility and invasiveness of cancer cells. In breast cancer, *Sukocheva, O et al *[55]demonstrated that E2-induced EGFR transactivation in human breast cancer cells is driven via a novel signaling system controlled by the lipid kinase sphingosine kinase-1 (SphK1). E2 stimulates SphK1 activation and the release of sphingosine 1-phosphate (S1P), by which E2 is capable of activating the S1P receptor Edg-3, resulting in the EGFR transactivation in a matrix metalloprotease-dependent manner. These findings reveal a key role for SphK1 in the coupling of the signals between three membrane-spanning events induced by E2, S1P, and EGF. However, it is still difficult to understand how such short-lived activation can be responsible for the profound involvement of SPHK1 in tumorigenicity or how this relates to its up-regulation in cancer. Our results imply that SPHK1 may be the central controller of amplification loops of EGF, AREG and EREG-EGFR interactions that can contribute to cancer progression.

## Conclusions

We have established an esophageal carcinoma invasion model and generated a highly invasive tumor cell subline in which *SPHK1 *was overexpressed. Further investigation revealed *SPHK1 *was significantly correlated with esophageal cancer invasion and metastasis and may be a valuable prognostic marker. Our studies have demonstrated that SPHK1 is involved in upregulation of EREG and AREG through enhancing EGFR phosphorylation to promote invasion. Thus, modulating SPHK1 expression or activity is an attractive additional therapeutic strategy for treatment of esophageal cancer and perhaps other cancers as well. Implementation of pre-clinical and clinical evaluation of SPHK1 as a novel molecular target for cancer therapy is warranted.

## Competing interests

The authors declare that they have no competing interests.

## Authors' contributions

PJ and NJ designed the study and wrote the manuscript, FX and ZWL participated in data analysis, PJ, WJ, ZYL and TYF finished the most experiment, HSY and WSY performed flow cytometry analysis. CBR and LZ collected the samples and made great contribution in making the tissue microarray. All authors read and approved the final manuscript.

## Authors' information

Pan Jian, Ph.D. Immunology. Graduated from State Key Laboratory of Molecular Oncology, Cancer Institute (Hospital), Peking Union Medical College, Chinese Academy of Medical Sciences, Beijing, PR China. Now is an associate professor of Department of Hematology and Oncology, Children's Hospital of Soochow University, Suzhou, China, and a gust professor of Translational Research Center, Second Hospital, The Second Clinical School, Nanjing Medical University, Nanjing, China.

## Supplementary Material

Additional file 1 **The gene expression profiles of EC9706 and EC9706-P4 were analyzed by microarrays**. Between these cell lines, 124 genes were differentially expressed, including 71 upregulated genes and 53 downregulated genes in EC9706-P4 relative to EC9706 (cutoff, fold ≥ 2.0). Click here for file
